# Assessment of the Push-Out Bond Strength for Glass Fiber Posts After Different Surface Treatments: An In Vitro Study

**DOI:** 10.7759/cureus.41499

**Published:** 2023-07-07

**Authors:** Akhila Raj. R, Fahiem Mohammad El-Shamy, Mannur Nikita Ajit, Kasturi Roy, Anjali Oak, Bilal Ameer

**Affiliations:** 1 Department of Conservative Dentistry and Endodontics, Amrita School of Dentistry, Amrita Viswavidyapeetham, Amrita Institute of Medical Sciences (AIMS), Kochi, IND; 2 Department of Dental Biomaterials, Faculty of Dentistry, Mansoura University, Egypt, EGY; 3 Department of Conservative Dentistry and Endodontics, SJM Dental College and Hospital, Karnataka, IND; 4 Department of Conservative Dentistry and Endodontics, Chandra Dental College and Hospital, Barabanki, IND; 5 Department of Conservative Dentistry and Endodontics, College of Dental Sciences and Research Centre, Ahmedabad, IND

**Keywords:** peeso reamers, ah plus sealer, surface treatment, silanization, glass fiber post

## Abstract

Aim: The goal of the study was to assess the push-out bond strength of the glass fibre post after different surface treatments.

Materials and methods: For the purpose of the investigation, 40 mandibular premolars were chosen. After gaining access, the biomechanical preparation was completed using the step-back approach up to a size 40K file, and the canals were sealed using gutta-percha cones and the lateral condensation procedure with AH Plus sealer (epoxide-amine resin pulp canal sealer). Peeso reamers were used to remove the canal fillings, leaving 5mm of gutta-percha apically. Drills included in the package were used to prepare the post spaces so that the posts would fit in their respective post slots. These were attached to self-curing acrylic resin blocks. Fibre posts were split into four groupings of n = 10 each for surface treatment, i.e., control, hydrogen fluoride, sandblasting, and hydrogen peroxide. The cementation of posts was done by utilising dual-cure resin cement. Two millimetres of the anatomical crown were removed from each sample. Each sample's 1-mm cervical segment was taken utilising the isotope from the remaining coronal area. To perform a push-out test, at the rate of 0.5mm/min of the crosshead, every sample was inserted into a universal testing device. Each post's dislodge force from the pre-set post spacing was measured. Statistics were utilised to analyse the data.

Results: Strongest bonds were made by silanization, followed by sandblasting (p value=0.002). The weakest bonds were made by the control group.

Conclusion: The ultimate deduction was that when glass fibre posts underwent various types of surface treatments followed by silanization, it had a significant impact on increasing their strength.

## Introduction

Due to substantial structural loss driven by caries and access cavity preparation, endodontically treated teeth need post-core restorations to serve as a retention mechanism [1]. Posts are often utilized to preserve a core for the final restoration and to repair teeth that have been treated endodontically and have a limited crown portion remaining [1]. It has been demonstrated that posts, rather than strengthening the tooth, improve the attachment between the manufactured crown and the residual structure of the root as well as distribute occlusal pressures aligned towards the longitudinal plane of the tooth [2,3]. The practitioners still use custom-cast posts and cores, which have been the gold standard for many years, to restore these endodontically treated teeth [4]. In order to rehabilitate teeth that had undergone endodontic treatment, cast or pre-fabricated metal posts were replaced with the advent of the glass fibre post technology [5,6]. The components of a glass fibre post are glass fibres embedded in a resin matrix, which is held together by a silane coupling agent [1,6]. The fibre posts are superior because their stress fields are almost the same as those of natural dentin because of their near proximity to dentin in terms of elastic modulus, which lowers the likelihood of fractures of the root [7]. For posts to be retained, both a strong connection between the cement and the canal walls and a close fit between the walls of the root canal and the post are required [1,6,8]. According to reports, the most frequent reason for their failure is debonding. As a result, bond strength is crucial for clinical purposes [6,9,10]. Bond failure of a post-core restoration, which occurs at the intersection of dentin and resin cement and accounts for roughly 60% of all defects [11], is the most major etiology of adhesive failure. The robustness of the attachment at the post and cement junction can only be guaranteed by the molecular reactions involving the fibre post exterior and the resin cement [7].

Restorations utilizing glass fibre posts survive longer [12-15], and restoration longevity is greatly influenced by the post's and resin cement's ability to adhere to dentin [13]. The strength of the bond between resin materials and posts appears to be influenced by mechanical as well as chemical treatments applied to glass fibre post surfaces [14,15]. Therefore, a strong and efficient bonding is critical among a resin cement as well as a fiber-reinforced system. The surface energy of fiber-reinforced posts has been enhanced through the development of various protocols for mechanical as well as chemical surface treatment [14,16-18].

No additional treatment is required prior to silane application for the purpose of chemically strengthening the bonding between a resin cement and the post [19,20]. To improve the micromechanical or chemical type of retention at the junction between the resin cement and post, other surface treatments must be performed prior to the application of silane, as investigations have demonstrated that only silane as a sole entity doesn’t enhance the strength of the bond of fibre posts with resin-based cement [21-23].

While other surface-treatment options have been explored, researchers are divided on whether silanization actually improves glass fibre post retention [19,22]. By removing the epoxy resin matrix layer and expanding the region in association with the fibres that will be silanized, roughening the post surface using mechanical or chemical techniques has been demonstrated to increase the survival of glass fibre posts using resin cement as a luting agent [24,25].

Binding between resin cement and fibre posts may be enhanced via surface therapy. The fibres of this post's epoxy resin are exposed due to these surface alterations, which also increase the surface area and roughen the fibre post's exterior [22,26]. This thus offers more retentive sites for the luting agent's micromechanical retention [22].

Nevertheless, despite laboratory studies on glass fibre post-surface therapies, a universally accepted protocol does not exist in order to obtain the best quality of adhesion. In order to assess the push-out bond strength of the glass fibre post after different surface treatments, this research set out to examine those effects in an in vitro environment. The aim of this study was to evaluate the push-out bond strength of glass fiber posts after undergoing different surface treatments, including silanization, sandblasting, hydrogen fluoride, and hydrogen peroxide.

## Materials and methods

For the investigation, convenience sampling was done. Forty intact mandibular premolars were chosen, with the power of study as α=0.8. A specialized endodontist carried out the procedure. Access was gained, and the working length was determined using the No. 10K file. The step-back method was used to do biomechanical preparation until a size 25K file touched the working length. Up to file number 40K, the canal has been widened and formed. To obtain a smooth tapering shape for the root canal, the master apical file was used to refine the root canal using circumferential filing. Gutta-percha cones and AH Plus sealer were used to obturate canals using the lateral condensation approach.

After utilizing peeso reamers to remove the root canal fillings, 5mm of gutta-percha was left apically. The posts were then fitted into the relevant post spaces after making the necessary post space preparations using the kit's provided drills. Then they were put on blocks made of self-curing acrylic resin.

The 40 posts were split into four categories containing 10 posts per category, and the surface treatments were carried out in the following manner: Category 1: only silanization (control); Category 2: silanization and hydrogen fluoride; Category 3: silanization and sandblasting; Category 4: silanization and hydrogen peroxide.

**Figure 1:**
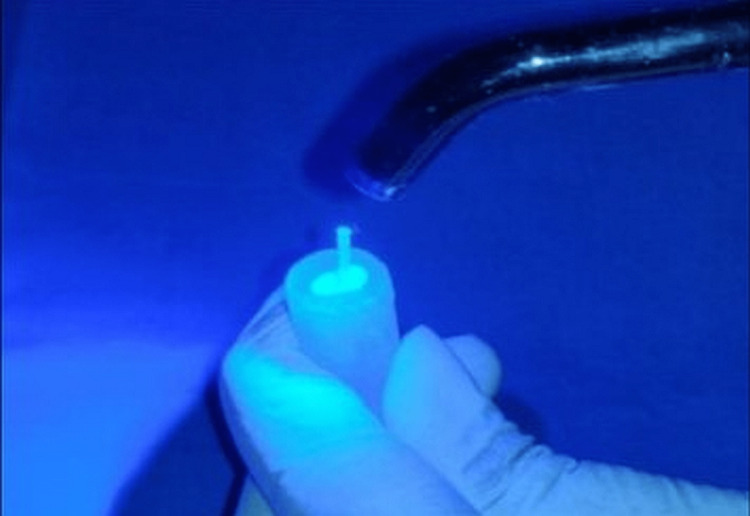
Cementation of the post in the post space

Then, saline was used to clean and treat the holes so they were ready for the posts. Paper points were employed for making the canals dry. A resin cement has been employed to lute the posts into the canal after they had been etched and a bonding agent had been applied. All examples had anatomical crowns that were decoronated to a depth of 2mm (Figure 1).

**Figure 2:**
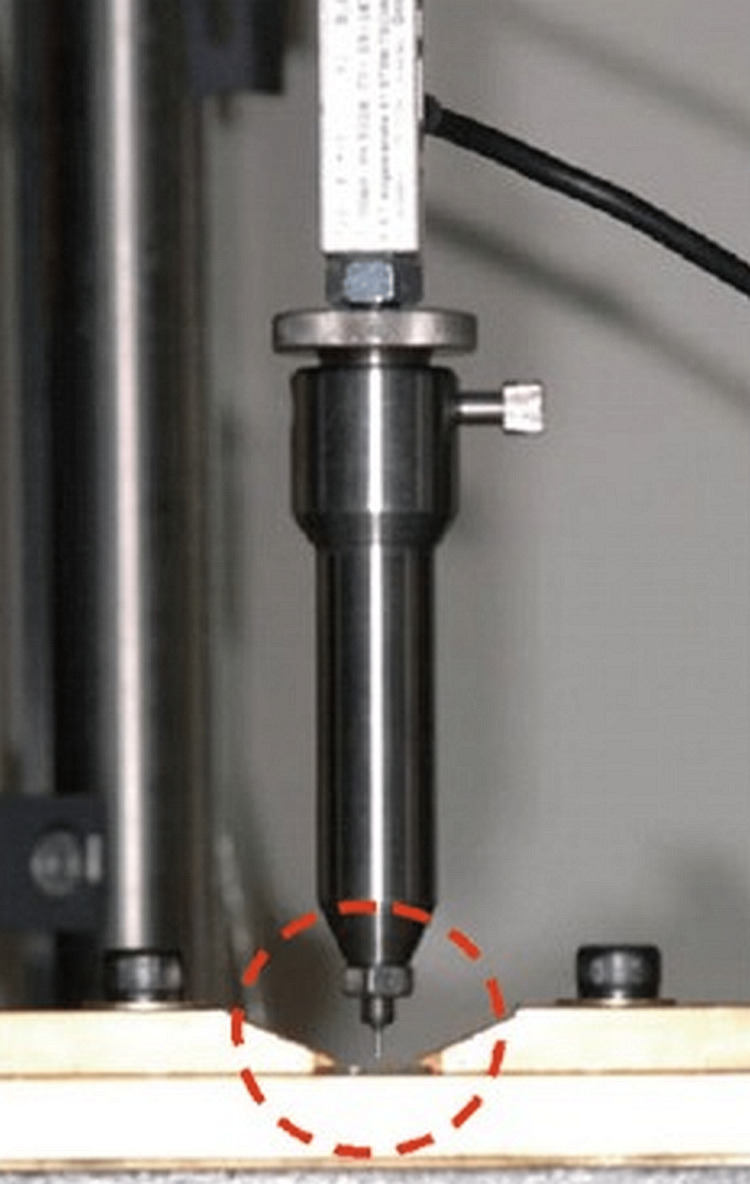
Push bond strength testing machine Circular region: where the testing is occurring

Each sample was cut into a 1mm-thick cervical segment using the isotope from the remaining coronal area. Each of these samples underwent a push-out test utilizing a universal testing device (Tinius Olsen, Horsham, PA, USA) whose crosshead speed was 0.5mm/min. Every post's dislodgement force from the pre-set post spacing was measured. The same operator carried out the evaluation of results (Figure 2).

Statistical analysis was then performed on the data [8-10]. Scheffe test was used for the analysis of variance (ANOVA) to test multiple comparisons of mean values amongst groups. The a-level of significance was 0.05 for all testing. All analyses were performed using SPSS 16.0 (SPSS GmbH, Munich, Germany).

## Results

According to statistical analysis, the post-surface treatments considerably affected the push-out bond strength of the fiber posts that underwent surface modification. More specifically, compared to the group treated with hydrogen peroxide, the control group, and hydrofluoric acid gel, the bond strength obtained after treating the glass fiber posts with sandblasting and silanization was much greater (p=0.003). Bond strength was lowest in the control group and this was quite significant (p<0.05) from the point of view of statistics (Table 1, 2).

**Table 1:** Comparison of push-out bond strength levels for fibre posts luted with dual cure resin cement based on diverse surface treatments SD: standard deviation, N: number of posts, HF: hydrofluoric acid, H2O2: hydrogen peroxide

Chemical	SD	Mean	N	F	p value	
						Silane	1.4	3.1	3	13.02	0.002	
HF	2.0	8.6	3								
Sandblasting	2.1	11.3	3								
H2O2	1.1	5.5	3								

**Table 2:** Scheffe Multiple Comparisons amongst groups

Pair of groups	F	P value
Group 1 and Group2	5.09	0.029
Group 1 and Group 3	11.47	0.003
Group 1 and Group 4	1.03	0.431
Group 2 and Group 3	1.28	0.346
Group 2 and Group 4	1.54	0.276
Group 3 and Group 4	5.63*	0.023

## Discussion

As radicular dentin is said to adhere more erratically than coronal dentin, the bond's quality may be considerably diminished and susceptible to deterioration. Adhesion is more difficult in apical dentin than coronal dentin as a result of morphological variations in radicular dentin. Microscopic analyses have disclosed that the coronal third of the root canal has increased resin tags as compared to the middle and apical third because the cervical third's tubule density is substantially greater [27,28]. As a consequence, it was determined that comparing cervical sections across all samples would provide more accurate findings than comparing middle or apical sections. However the limitations of push-out tests include the following: Push-out test when performed on thick root sections or on whole post causes non-uniform shear stress distribution. The specimen position, the angle at which the load is applied influences the push-out bond strength results. To overcome these limitations the specimens were modified in our study to obtain 2mm thick dentin slices.

In teeth that had been through endodontic treatment and were then restored using post-and-core systems, post-debonding or decementation showed out to be the most common form of failure, while fractures of the root in the vertical direction had been the most significant kind of disappointment [29].

Unlike posts that are normally bonded, adhesively luted posts stay in place better, which may lead to less debonding. The adhesive is used to lute glass-fibre posts into the root canal. Additionally, the bonding of posts has been shown through finite element analysis to significantly reduce stresses inside the root canal; as a result, this should be a key factor in avoiding fractures of the root [30]. No chemical bonding can be anticipated between the epoxy resin matrix of pre-fabricated fibre posts and the resin matrix, which is primarily methacrylate-based, due to their different chemical compositions [31]. A number of chemical treatments have been explored for the purpose of increasing the available surface area of the fibre posts' epoxy or methacrylate-based resin matrix and exposing the glass fibres as well as their filler particles so that they may be accessed by the surface chemical modifiers.

Chemical treatment techniques that first give micromechanical retention and then bond chemically with a silane coupling agent can make the posts stick better. The three main types of surface therapy for the post are as follows: surface roughening (i.e., acid etching and sandblasting), chemical treatment (i.e., silane application), or a combination of the two [32]. Quartz fiber posts' binding strength can be increased by using a silane coupling agent, according to one study, while the effect on glass fiber posts was less clear [31]. In the present study, there was a significant difference observed between the silane group (Control group) and the sandblasting group (Group 3). 

Chemical bonding to fibre posts

The wetting capacity of the silane is recognised as playing a critical role in improved adhesion according to the theory of surface wetting developed by Pape and Plueddemann. As per this hypothesis, the low viscosity of silane would help the substratum to remain moist, and once a close connection between the interjunction of materials is made, van der Waals forces would start to work, creating a physical adhesion that aids in the chemical process [33]. The glass-covered substrate can form a chemical bond with the silane coupling agents' ability to enhance surface wettability [34]. With the use of a silane coupling agent alone, the achieved strength is relatively modest. This might be a result of resin compounds and the matrix of fibre posts not chemically uniting [35]. The degree of conversion and cross-linking in epoxy polymers is greater. Amino silane coupling agents are frequently used for promoting adhesion after surface changes occur due to the existence of epoxy resin polymers in the fibre posts [36]. The process of silane coupling requires a very precise technique. Its effectiveness depends on its makeup and method of application. Small amounts of solvent can improve silane wetting, and while its incomplete removal can impair coupling, solvent evaporation is crucial [37]. To obtain the best reaction, it may be triggered by heating or treating with acid, which could then be accompanied by adding a coupling agent such as silane.

The chemical preparation known as silanization is the most popular. Bifunctional molecules called organo-silane coupling agents can combine with inorganic glass fibre on one end while interacting with resin cement on the other [38]. However, there is debate over the function of silanes in the bonding of resin luting agents to glass fibre posts. According to a few investigations [39-41], silanization doesn't really significantly affect the adhesion strength among both resin cements and glass fibre posts, while silanization was found to enhance bonding in other studies [42,43]. There is also a chance that increasing the glass fibres' exposure to physical or chemical pretreatments will have a positive synergistic impact on silanization, enhancing the survival of glass fibre posts [44].

Micro-mechanical and chemical bonding to fibre posts

Surface treatment, which makes it easier for various elements to be retained chemically and micromechanically, is a typical technique for enhancing a material's overall adhesion qualities. Here are the several conditioning techniques that were evaluated on fibre posts.

Hydrofluoric Acid (HF)

This group utilised etching with 9.6% HF gel and performed second best, with superior push-out bond strength than those of the control and hydrogen peroxide groups. The purpose of etching with hydrofluoric acid was to roughen the surface to enable micromechanical interaction with the restoration. Acid has a time-dependent impact. This method seriously compromised the structure of the post and severely damaged the glass fibres [45]. After this treatment, the glass strands seemed to be less durable. This is because hydrofluoric acid has a very corrosive impact on the postmaterial’s glass phase. Upon the usage of hydrofluoric acid for conditioning the fibre posts, a remarkable surface change was noticed along with the increase in bond strength. As a result, it is impossible to recommend a comprehensive policy for using hydrofluoric acid [46]. Due to its caustic character, hydrofluoric acid etching, which creates microspaces between the exposed fibres, is used to treat the surface of fibre posts [47]. Although this surface treatment produces satisfactory bond strength outcomes, utilisation of this particular acid can be harsh on the post fibres. Usage of hydrofluoric acid for the purpose of etching encourages invasive alteration of the post despite increases in bond strength by impacting the adjacent epoxy resin and damaging the fibres, which reduces the mechanical properties of the posts [48]. Along with the potential for these harms, this type of treatment of the exterior surface exhibits a lesser strength of the bond when contrasted with different synthetic or chemical agents [49]. The protocols that are available do not conform to any particular standards, neither in concentration of acid nor time given for application, and this may be the cause of variation. Consequently, it is best to avoid using such potent acidic solutions to avoid harming the strands [50].

The integrity of the post was affected by this approach, which severely damaged the glass fibres [45]. Despite the bond strength gains, HF etching induces aggressive post modification by interacting with the surrounding epoxy resin and damaging the fibres, which reduces the mechanical strength of the posts [48]. Compared to other chemical treatments, this surface treatment has poorer bond strength outcomes in addition to the potential for significant damage.

Hydrogen Peroxide (H202)

By removing an epoxy resin surface layer, surface modification of glass fibre posts with H2O2 enhances the surface area subjected to silanization and provides positions for the retention of the luting agent by forming a micromechanical bond [51]. The impact of etching depends on hydrogen peroxide's capacity to partly break down the resin matrix and rupture the links between epoxy resin molecules through substrate-level oxidation [52]. It is crucial to keep in mind that all therapies with H2O2 reveal the fibres without harming them. The electrophilic assault of H2O2 on the dried secondary amine is likely what causes the epoxy resin to dissolve [24]. As a result, the spaces between the fibres produce the necessary circumstances for the resin adhesive and post to micromechanically lock together. Without using excessively caustic solutions in a clinical context, H2O2 etching offers a simple, efficient, and practicable way to improve the interfacial strengths between posts and cement [31]. Additionally, the silane agent makes it possible for the exposed fibres to chemically bind to the adhesive. 

There is evidence that the use of peroxides may make it harder to glue posts in place during endodontic treatments [24]. It is thought that this is because there is still oxygen in the tubules of the dentin, which stops the adhesive resin from hardening [53]. However, applying peroxide to the fibre staple strengthens the bonds. Due to the lack of residual oxygen in the post structure, the peroxide's harmful impact was probably not noticed [24].

Sandblasting is often used in general industry to roughen the surface of things, which makes them easier to stick together. It is often used in tribochemical silica-coating processes, indirect composite bonding, metal-ceramic restorations, ceramic and composite repair methods, and pretreatment of metal surfaces [54]. In particular, it is often used in adhesive dentistry to prepare materials that are resistant to acid. It can make the surface of a restoration more uneven, giving it a honeycomb-like appearance, which is anticipated for the retention of micromechanical type [55]. Sandblasting the surface has greatly increased bond strength, which is consistent with the results of earlier investigations [48]. It is seen as an aggressive procedure since the sandblasting process might significantly alter the post's form and volume. The fibre post's surface was made rougher during the sandblasting process, which caused the resin cement and fibre post to mechanically bond. A larger bonding surface area can also be formed by the increased roughness. The results of sandblasting are influenced by the duration of application, the pressure applied, and the distance between the sandblasting unit and the fibre post surface [9].

Sandblasting causes the post surface to become rougher by eliminating the matrix made of resin between the fibres of silicon, thus enhancing its retentive capacity and increasing bond strength in comparison to other groups. Additionally, the chemical reaction of silanes, which relies on the synthesis of bonds of siloxane and the transformation of the mineral surface into a less polar surface compatible with an organic bonding agent, also depends on the conversion of the surface of the mineral [56]. Sandblasting and silanization, according to Wang et al., increase the strength of the bond between resin cement and fibre posts. Before being used in clinical environments, they suggested that fibre posts should be treated prior to usage with sandblasting or combined with silanization [57].

Acid etching is commonly used to create micromechanical retention on the post's surface. A study by Santos et al. [30] evaluated the effect of acid etching on the push-out bond strength and found that it significantly increased the bond strength compared to untreated posts. Conversely, Lewis et al. [29] reported no significant difference in bond strength between acid-etched and untreated posts. These conflicting findings warrant further investigation and standardization of acid etching protocols.

Cementation works because of the strong and long-lasting bonds between the post-cement-root dentine complex and the root dentine. The root canal architecture, the amount of moisture present in the canal, the density and direction of the root dentinal tubules, as well as the affinity of the agent used for luting with the substratum and the polymerization of the cement used for luting, all play a role [9]. Resin cements of the dual-cure type have been recommended for lute fibre posts because light cannot guarantee sufficient copolymerization in the root canal's deep regions. Dual-cure cements' chemical reaction, though, can't entirely compensate for inadequate polymerization in dimly lit, gloomy environments [58]. The apical one-third and middle one-third showed lower strength of the bond formed than the cervical third, which showed a higher amount of it. Dual-cured resin cement yields more polymerization stress than self-cured cement, which results in interjunctional discrepancies that affect the effectiveness and authenticity of the adhesive interface. Self-cured cement has a longer polymerization process than dual-cured resin cement, which enables improved creep of the cement and lessens polymerization stress [59].

Silanization involves the application of silane coupling agents to the post's surface, enhancing the adhesion between the resin cement and the post. A study by Silva et al. [59] investigated the effect of silanization on the push-out bond strength of glass fiber posts and reported a significant increase in bond strength compared to untreated posts. Similarly, Oliveira et al. [43] observed improved bond strength after silanization, supporting its efficacy as a surface treatment method. As a result, the sort of luting agents and interface location also had an impact on the retention of the fibreglass post. A modest form of sandblasting (50mm Al2O3 particles, 2.0 bar, 10s, 5cm) was recommended by D'Arcangelo et al. to treat the surface of fibre posts and improve mechanical retention without reducing their flexural capabilities [47]. According to certain researchers Wrbas et al. [60] and Aksornmuang et al. [61], the bonding effect on fibre posts can be greatly improved by the silane coupling agent. In a study by Akhavan et al. [62], different laser treatment modalities were compared, and it was found that Er:YAG laser irradiation significantly increased the bond strength. In contrast, Nd:YAG laser treatment did not improve the bond strength significantly. Further research is required to explore the optimal laser parameters and their long-term effects on the bond strength.

Thus, a number of variables affect how a post and resin cement interact, including the exposure of fibres due to treating the exterior of the post with chemicals and the resin cement's interlocking with post surface micro-spaces that increase retention.

The study has limitations with the number of sample sizes, and it has also checked a few of the chemical properties, which can be checked for multiple times in future research. To validate the in vitro finding and settle conflicting data in the already published literature, a clinical study of these concepts is required.

## Conclusions

The investigation's findings showed that surface alterations to the fibre post’s exterior considerably altered its push-out bond strength. Therefore, under the constraints of this investigation, it has been concluded that the optimal surface modification procedure for glass fibre posts was sandblasting for 20 minutes followed by silanization, as opposed to H2O2, HF, and the control group. This in vitro research does not predict whether the fibre posts' in vitro and in vivo performance will be identical.

To determine whether the beneficial impact on the strength of the bond between the post and the built-up core still exists after pre-treatment of the surface of the post much before it’s a clinical application, additional ex vivo as well as in vivo research must be done. Manufacturers will be able to provide pretreated fibre posts in sealed sachets as a result of this strategy's evaluation, saving clinicians precious chair time. For an effective restoration, additional factors like flexural strength, fracture resistance, and microleakage must be assessed. The findings of the current study must be confirmed by additional research on these fibre-post devices.
